# Multiple neural states of representation in short-term memory? It’s a matter of attention

**DOI:** 10.3389/fnhum.2014.00005

**Published:** 2014-01-23

**Authors:** Joshua J. LaRocque, Jarrod A. Lewis-Peacock, Bradley R. Postle

**Affiliations:** ^1^Medical Scientist Training Program and Neuroscience Training Program, University of Wisconsin-MadisonMadison, WI, USA; ^2^Department of Psychology and Institute for Neuroscience, University of Texas at AustinAustin, TX, USA; ^3^Department of Psychology and Department of Psychiatry, University of Wisconsin-MadisonMadison, WI, USA

**Keywords:** short-term memory, attention, representational states, multivariate pattern analysis, unattended memory items

## Abstract

Short-term memory (STM) refers to the capacity-limited retention of information over a brief period of time, and working memory (WM) refers to the manipulation and use of that information to guide behavior. In recent years it has become apparent that STM and WM interact and overlap with other cognitive processes, including attention (the selection of a subset of information for further processing) and long-term memory (LTM—the encoding and retention of an effectively unlimited amount of information for a much longer period of time). Broadly speaking, there have been two classes of memory models: *systems models*, which posit distinct stores for STM and LTM (Atkinson and Shiffrin, 1968; Baddeley and Hitch, 1974); and *state-based models*, which posit a common store with different activation states corresponding to STM and LTM (Cowan, 1995; McElree, 1996; Oberauer, 2002). In this paper, we will focus on state-based accounts of STM. First, we will consider several theoretical models that postulate, based on considerable behavioral evidence, that information in STM can exist in multiple representational states. We will then consider how neural data from recent studies of STM can inform and constrain these theoretical models. In the process we will highlight the inferential advantage of multivariate, information-based analyses of neuroimaging data (fMRI and electroencephalography (EEG)) over conventional activation-based analysis approaches (Postle, in press). We will conclude by addressing lingering questions regarding the fractionation of STM, highlighting differences between *the attention to* information vs. *the retention of* information during brief memory delays.

## Introduction

Understanding the neural mechanisms that support the short-term retention of information is a long-standing aim of cognitive neuroscience. For many years, the landscape of memory research was dominated by *systems-based models* that emphasized a division between primary and secondary memory, with the former corresponding to short-term memory (STM) and the latter to long-term memory (LTM). Baddeley and Hitch (1974) codified the idea of a system of distinct STM storage buffers in their multiple component model (for a recent depiction, see Baddeley, 2003). These buffers operate on the principle of the temporary activation of mental representations (as distinct from the “passive trace” of LTM). The idea of elevated activity as the basis for STM also has a long history in neuroscience, dating back at least to Hebb (1949), and explicitly proposed by Goldman-Rakic and colleagues (Funahashi et al., 1989) as a neural implementation of the STM buffers of the multiple component model.

More recently, many researchers have recognized that a system of STM buffers supported by an active trace cannot parsimoniously accommodate the growing evidence for tight links between attention, STM, and LTM (Ruchkin et al., 2003; Ranganath and Blumenfeld, 2005; Postle, 2006). Newer *state-based models* of STM have proposed that interactions between attention and LTM may act as the basis of short-term retention (Cowan, 1995; McElree, 1998; Oberauer, 2002). These models conceptualize information in STM as existing in various states of activation established by the allocation of attention. This review will highlight how contemporary cognitive neuroscience research is broadly consistent with these state-based models of STM, and consider how the two literatures inform one another.

## Short-term memory (STM): theoretical models and behavior

**Figure 1:**
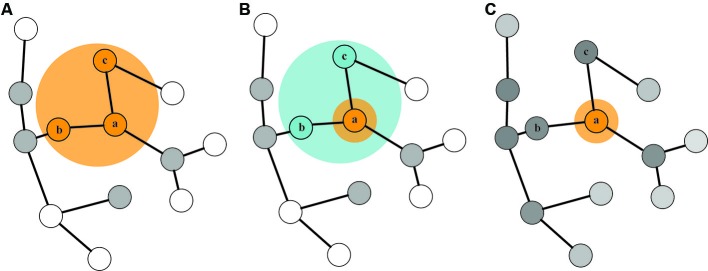
**Three state-based models of STM**. In each of the three models, the basis of STM representations is a network of LTM. In (Cowan’s 1995) model **(A)** a capacity-limited FoA selects up to four items for further processing. Information that was recently selected by the FoA remains activated (denoted by the gray-colored nodes), and is termed the activated portion of LTM. Oberauer’s (2002) model **(B)** includes a narrow FoA which can efficiently select information (item *a*) from a highly activated region of direct access (containing items *b* and *c*; we refer to this region of direct access as the “state of direct access” (SDA) in this manuscript). After information becomes deprioritized, it transitions to a state of activated LTM. McElree’s (1998) model **(C)** posits a single-item FoA (containing item *a*), with all other memory in a common state with smoothly varying levels of memory strength.

First, we will summarize three prominent theoretical accounts which postulate that information in STM can be retained in different states as defined by the interactions between attention and LTM (Figure 1; Cowan, 1995; McElree, 1998; Oberauer, 2002). Cowan (1995) describes two distinct states in STM: a small, capacity-limited state referred to as the focus of attention (FoA) and a more expansive state referred to as the activated portion of LTM (aLTM). In this model, the FoA corresponds to approximately four chunks of information that one can hold in STM using top-down attentional control at any moment in time. When attention subsequently shifts to other information, the items that were previously in the FoA transition into aLTM. aLTM has no capacity limit *per se*, but is susceptible to temporal decay and interference effects. A modification of this view was proposed by Oberauer in the three-embedded-components theory (Oberauer, 2002, 2009). In this view, the four-item FoA from Cowan’s model is recast as a *region of direct access* from which a narrower FoA can efficiently select information. Capacity limits, *per se*, do not exist for either of these two hypothesized states in STM. Rather, the amount of information that can be retained in the direct-access region and the FoA is limited only by interference from bindings between object features being retained in STM (Oberauer, 2013). A third model, advocated by McElree (1998, 2006), posits two components of STM: a FoA with a strict capacity limit of one item, and LTM, in which items are equally accessible, but can differ in terms of “memory strength”. This model also allows for a representation of how recently an item was in the FoA, a property that we will equate to “activation” as defined in relation to the other two models.

Although many of the ideas espoused by these models are consistent, the terminology often is not. What Cowan calls the FoA is called the direct-access region in Oberauer’s model (or, more recently, the “broad focus” (Oberauer and Hein, 2012)). Oberauer’s FoA has no direct equivalent in Cowan’s model, although it is similar to McElree’s single-item FoA. In this review, we will use the conventions of Oberauer’s model, with the exception that we will refer to his “region of direct access” as a “state of direct access” (SDA), to avoid confusion with anatomical “regions” of the brain.

The three models summarized here have been developed to explain extensive behavioral findings (reaction times and accuracies on tests of memory) suggesting the existence of different states of representation in STM. For example, Oberauer (2001, 2002, 2005) has made clever use of the Sternberg effect, whereby reaction time (RT) for a recognition judgment about a memory probe increases linearly with the number of items concurrently held in memory. The Oberauer studies modified the basic Sternberg memory paradigm by cuing the subject during a brief memory delay that only a subset of the initially presented memory items would be relevant for an upcoming probe. Given sufficient time to react to these “retrocues”, subjects respond more quickly to memory probes of the cued items. The uncued items are not fully forgotten, however, and continue to influence ongoing processing in the form of an intrusion effect on response times (that is, slower response times) when they are presented as negative (to-be-rejected) memory probes. This intrusion effect persists for 5 s, long after the uncued items cease to affect response times for the cued items. These items are hypothesized to have been removed from the SDA into aLTM (Oberauer, 2001). The existence of multiple states of STM in Oberauer’s model, and the dynamic transitions between these states, were inferred primarily from this set of results. Moreover, by varying the retrocue-to-memory probe asynchrony it has been estimated that it takes ~1 s to remove uncued items from the SDA (Oberauer, 2005).

And what about the fate of the uncued memory items? A recent study examined behavioral accuracy for probes to memory items (in this example the memory items were colored circles) that were not prioritized by a retrocue during the first portion of the trial (Rerko and Oberauer, 2013). The authors used a design in which two different retrocues appeared on each trial, serving to sequentially signal the relevance of two different items in memory—subjects knew that only the item indicated by the last retrocue would actually be the target of a memory probe. The recognition probe appearing after the second of these cues therefore probed a memory item that was initially uncued. (There were also trials with only a single retrocue followed by a probe; the presence of these trials ensured that subjects indeed allocated their attention to the item indicated by the first retrocue). Behavioral accuracy on the two-cue trials was statistically indistinguishable from trials with only a single cue, suggesting that there is no loss of memory strength for memory items that temporarily transitioned out of the FoA after the initial retrocue. (We note, however, that the interval between the first and second cue onsets was only 600 ms; given results from our own behavioral and neural studies (LaRocque et al., 2013), this amount of time may not be sufficient for an item to transition out of the FoA before the onset of the second cue).

In contrast to these findings, which provide support for multiple-state models of STM (Cowan, 1995; Oberauer, 2005), other studies have suggested that STM comprises only one distinct component, the FoA, within a network of memory that is not qualitatively different from the myriad memories stored in LTM (McElree, 1998, 2006). In this formulation, the emphasis is on *memory strength* and *retrieval speed*. The studies in support of this view have employed behavioral tasks with serially presented memory items, under the assumption that the last item presented before the memory probe would be maintained in a FoA (McElree and Dosher, 1989). By playing a tone to signal when subjects could respond to the memory probe, the authors attempted to control speed-accuracy trade-offs that are omnipresent in behavioral paradigms with speeded responses. In these experiments, the memory strength (measured by the asymptotic accuracy reached at long probe-tone intervals) decreased monotonically as a function of the serial position at which the probed memory item was presented. For example, in a six-item trial, the second item presented had lower memory strength than the fifth item according to these measures of response accuracy. In contrast, the retrieval speed, measured by the rate constant of the fit between accuracy and response time at varying probe-tone intervals, was the same across all memory items except for the most recent item. From this pattern of results, McElree has argued that only the last item in the list of STM items can be held in the FoA; all other items are maintained as discrete “events” in LTM with varying levels of memory strength, while sharing identical retrieval dynamics. In McElree’s words, “[R]etrieval from what is traditionally thought to be working memory (WM) is mediated by the same mechanism that is typically argued to underlie retrieval from LTM” (McElree, 2006).

To summarize, state-based models posit that STM performance is accomplished via the activation of LTM representations, with information that is “in mind” being held in a FoA, and recently attended items having a level of activation that is greater than the basal level of information in LTM. Two important points where these models diverge is on the capacity of the FoA (*-1* item vs. many), and whether or not there is a qualitative distinction between states in STM outside the FoA and the remainder of LTM. We will return to these questions near the end of this review, to consider whether the neuroimaging findings that are the focus of this review might help adjudicate between these different theoretical propositions.

## Neural activation and representational states in short-term memory (STM)

We preface this section by noting that fMRI has been used to validate the general idea that STM can be supported via the temporary activation of LTM representations: a multivariate classifier trained to discriminate three categories of information from LTM can decode the category of information being maintained during a test of STM (Lewis-Peacock and Postle, 2008). This study, however, did not address the distinct retention states proposed by the theoretical models reviewed above. The remainder of this review will consider more recent studies that have been explicitly designed to seek neural correlates of these different hypothesized states of representation in STM. We will categorize these studies by methodological strategy: those that assess the neural signal associated with the memory probe, and those that assess the neural signal associated with the delay-period during tests of STM.

### Probe-based neuroimaging studies

The empirical studies reviewed in the Section Short-term Memory: Theoretical Models and Behavior all relied on behavioral performance metrics on tests of STM as proxies for the representational state of items being held in STM. In addition to examining response times to memory probes, neuroimaging studies can make inferences about representational states by evaluating probe-evoked neural signal. For example, Nee and Jonides (2008) used a paradigm in which subjects were presented with a series of three words followed 300 ms later by a recognition probe. They reasoned, like McElree (2006), that probes matching the most recently presented word (the “-*1* item”) would test subjects’ memory performance for an item held inside the FoA, whereas probes matching earlier stimuli (“-*2*” and “-*3*” items) would test their memory performance for items held outside the FoA (thus, probes of these items would require retrieval of these items back into the FoA). They found that the blood-oxygen-level-dependent (BOLD) response in the anterior inferior temporal cortex was greater for probes matching the *-1* item than for probes matching the -*2* and -*3* items. They also found increased activation of the medial temporal lobe (MTL) and left mid-ventrolateral prefrontal cortex (vlPFC) for probes matching the -*2* and -*3* items, compared to probes matching the -*1* item. In a more fine-grained distinction, they found enhanced functional connectivity between inferior temporal cortex and both right vlPFC and left posterior parietal cortex for probes matching -*1* items vs. probes matching -*2* items. This difference was interpreted as a neural dissociation between the retrieval of items inside vs. outside the FoA in STM. Later work from the same group replicated and extended this result by expanding the list of items to be remembered on each trial from 3 to 6 items (Nee and Jonides, 2011, 2013). The authors attempted to dissociate three putative states of STM by comparing neural activity evoked by probes targeting items presented at different serial positions in the list: the FoA (the -*1* item), the state of direct access (the -*2* and -*3* items), and the activated portion of LTM (the -*4* and -*5* items). Their results highlighted brain regions whose probe-evoked activity differed between these different probe types (Nee and Jonides, 2011). Left posterior superior temporal gyrus, left posterior parietal cortex, and left anterior inferior temporal cortex were associated with retrieval of information from the FoA (findings in the latter two regions replicated previous work (Nee and Jonides, 2008)). Right hippocampus was associated with retrieval from the SDA (previously, left parahippocampal and entorhinal cortex was associated with retrieval from the SDA (Nee and Jonides, 2008)). Finally, left inferior frontal gyrus was associated with retrieval from aLTM. Despite the compelling triple dissociation in probe-evoked activity, these data provide only indirect evidence for distinct “states of representation” in STM, because they do not measure neural signals associated with the retention, *per se*, of information in these hypothesized states of STM.

In contrast to these results, other probe-based studies have failed to find evidence for an SDA. Oztekin et al. (2010) collected fMRI data from subjects performing an STM task for 12 serially presented items, using the same logic as before to assume that the most recently presented item was in the FoA (Oztekin et al., 2010). Their primary question was whether the hippocampus, as a marker of LTM retrieval (Talmi et al., 2005), would show increasing probe-evoked activity for items at early positions on the stimulus presentation list. Because those earlier-presented items could be considered to be retained outside Oberauer’s SDA, the authors envisioned that earlier-presented items might therefore be in LTM and more reliant on the hippocampus for retrieval. In contrast to that scenario, they found that activity in the hippocampus was actually higher for probes to -*2*, -*3* and -*4* items, which were presumably in the SDA, compared with probes to -*5* through -*11* items. The authors ascribe two interpretations to these results: (1) retrieval from WM as well as LTM requires MTL activity, suggesting a common store; (2) MTL activity may be a better metric of memory strength than of LTM access, *per se*. Intriguingly, no brain regions showed increased probe-evoked activation for the early list items compared with the -*2*, -*3* and -*4* items. Most notably, they did not replicate the Nee and Jonides (2011) finding of heightened left inferior frontal gyrus activity for this contrast. However, the evoked activity from probes to the -*2*, -*3* and -*4* items was significantly higher than activity evoked by probes to early-list items in the left supramarginal gyrus (Oztekin et al., 2010); this result agrees qualitatively with the pattern observed in Nee and Jonides (2011), wherein probe-evoked activity in the left supramarginal gyrus can be seen to decrease with probes of items putatively in the FoA, the SDA, and aLTM, respectively.

From this summary, it is clear that the pattern of findings in the “probe-based” literature is inconclusive with regard to the theoretical models that motivated them. Additionally, we believe that this type of design suffers from shortcomings that limit, *a priori*, the inferences that it can support. Namely, the “probe-based” approach relies on the underlying assumption that probes matching items putatively stored in distinct representational states should evoke differential BOLD activation. As we noted earlier, such activity is not a direct measure of neural representations, *per se*, in STM. Furthermore, such differences in probe-evoked activity are not specific to one stimulus type, leaving open the possibility that the observed differences may be due to some factor other than stimulus representation, such as task difficulty or effort. A final note about these experiments is that the observed neural signals can be interpreted as reflecting two separate factors: (1) the *reorienting of attention* to the information being probed; and (2) the *decision process* required to respond accurately to the probe. We will now discuss a second category of neuroimaging studies, those designed to measure the state of activation of mnemonic representations during the memory delay, when the neural signal would presumably be less susceptible to contamination by other cognitive processes.

### Delay-based neuroimaging studies

#### Univariate analyses of different states in short-term memory (STM)

Intimately related to the debate over representation states in STM is the question of what might differentiate representations in STM from perceptual representations. After all, recent theoretical accounts have posited that the representational substrate for information in STM may reside in sensory cortices (Pasternak and Greenlee, 2005; Postle, 2006), and it is unequivocal that neuronal mechanisms necessary for representing stimulus information can be found in sensory cortices. Though theoretical models typically ascribe STM representation to the activation of previously latent LTM resources, a similar argument can be made for perceptual representations (Fuster, 2003). Nobre et al. (2004) have addressed this question by examining the neural differences between allocating attention to external stimuli vs. allocating attention to information already being held in STM. They used a task paradigm in which the location of a relevant memory item was provided via a spatial cue, which could be delivered either before (precue) or after (retrocue) the memory items were presented (Griffin and Nobre, 2003). Whereas the precue would allow for the pre-allocation of spatial attention in anticipation of the display of the memory items (externally oriented attention), the retrocue, occurring *after* stimulus presentation, would require attentional allocation to items retained in STM (internally oriented attention). The analysis of electroencephalography (EEG) data recorded while subjects performed this task revealed that the cue-evoked event-related potentials (ERPs) in both conditions revealed a common marker of attentional allocation: a negative deflection (the N1 component) in voltage recordings from posterior electrodes, greater in magnitude in the electrodes contralateral to the attended spatial location than in ipsilateral electrodes. In comparing the retrocue and precue trials, an ERP difference extending from frontal to posterior electrodes was observed between 200 and 400 ms after the presentation of the cue. This was interpreted as reflecting the engagement of additional frontal cortical regions when the target of attentional allocation was an internal representation vs. an external stimulus. Subsequent fMRI studies confirmed this result by finding enhanced activity in dorsolateral prefrontal cortex and parietal cortex for retrocues vs. precues (Nobre et al., 2004). Two primary conclusions can be drawn from these results. First, the similar ERPs over posterior electrodes for internally and externally oriented attention suggest a common site of the allocation of attention. This is in agreement with the suggestion that STM representations and perceptual representations might rely on the same cortical regions (specifically, sensory cortex). Second, the engagement of additional prefrontal and parietal cortical regions for internally directed attention vs. externally directed attention suggests that those regions’ roles might be specific to manipulating representations in STM. These regions are highly overlapping with those identified by many previous studies of neural activity during STM tasks (D’Esposito et al., 2000; Curtis and D’Esposito, 2003). The observation that these regions are preferentially active during shifts of attention to items in STM provides a possible explanation of the function of activity in these regions in previous studies of STM; namely, that it may also reflect the allocation of attention to representations in STM.

However, for questions regarding the allocation of attention to individual items in memory, these results from Nobre et al. (2004) are limited because they collapse across all stimuli; therefore, their description of attention allocation is necessarily a generalized one. A follow-up study attempted to address the question of how attention to a specific representation in STM might be manifested in neural activity (Lepsien and Nobre, 2007). The difficulty to be overcome by this study was how to assay neural activity related to allocating attention to one of two stimuli in STM. To do so, the authors took advantage of early findings of a different neuroanatomical loci of neural activity during the viewing of pictures of houses vs. faces: specifically, neural activity in the fusiform gyrus is higher when viewing pictures of faces than pictures of houses, and the opposite pattern is seen in the parahippocampal gyrus (Mccarthy et al., 1996; Kanwisher et al., 1997; Aguirre et al., 1998). Subjects were tasked with remembering two stimuli on each trial, one house and one face. After the pictures disappeared from the display, a cue appeared indicating which item would be relevant for an upcoming memory probe. The neural activity in region of interests (ROIs) separately determined to be responsive to faces and houses increased (or decreased) when the cues indicated that the ROI’s preferred category was relevant (or irrelevant). This result can be taken as supportive of separate levels of activation corresponding to attended and unattended items in STM. It also is in agreement with the idea that regions engaged in the perception of a stimulus might also be involved in the short-term storage of that stimulus type.

More recently, a thematically similar follow-up study, using umbrella-like cartoon images as memory items and a retrocuing design, observed neural activity after a retrocue related to the set size of the uncued memory set (Trapp and Lepsien, 2012). This effect was found in posterior intraparietal sulcus. In contrast, delay-period BOLD signal in anterior intraparietal sulcus only showed a load effect on trials with an uninformative retrocue that selected all memory items as relevant. On trials with a retrocue signaling one item that would to be probed, there was no load effect in anterior intraparietal sulcus, whereas a load effect persisted in posterior intraparietal sulcus. This pattern of results led the authors to suggest a posterior vs. anterior division in intraparietal sulcus between mnemonic vs. attentional functions, respectively. This result is notable because it can be interpreted as a neural signature of STM representations after they have transitioned from the SDA into aLTM. Recent developments in neuroimaging data analysis, the focus of the next subsection, mean that the interpretations drawn from some of the studies reviewed in this subsection may need to be reconsidered.

#### Multivariate analyses of neural representations

The neuroimaging studies reviewed to this point attempted to infer the existence (or non-existence) of various representational states in memory, or their relation to perceptual representations, based on differences in BOLD signal magnitude or location. The premise of this reasoning is that if the activation state of a neural representation varied as a result of an experimental manipulation, the corresponding level of fMRI signal intensity would vary in a congruent manner. However, recent advances in our thinking about the multivariate nature of neuroimaging data sets has brought about a renewed appreciation for the fact that this “signal intensity-based” reasoning also supports only indirect inference with regard to assaying active neural representations (Postle, in press). An approach that is maximally sensitive to the information contained in patterns of neural activity should provide a more direct measure of neural representations (Tong and Pratte, 2012; Davis and Poldrack, 2013). Recently, several studies have attempted to characterize neural representations in different states of STM by using multivariate pattern analysis (MVPA; e.g., Haxby et al., 2001; Haynes and Rees, 2006; Norman et al., 2006). This approach provides more inferential power due to its greater sensitivity at detecting information in neural signals. This increased sensitivity is illustrated by studies that have found stimulus-specific patterns of activity in early visual cortex during the delay period of a visual STM task, despite the absence of elevated delay-period activity in the same regions (Harrison and Tong, 2009; Serences et al., 2009; Riggall and Postle, 2012; Han et al., 2013; Lee et al., 2013). Similar results have been reported for decoding the frequency of auditory memory items from primary auditory cortex (Linke et al., 2011). The sensitivity of MVPA is further exemplified by a recent study that used MVPA to demonstrate a lack of *specificity* in univariate analyses of the BOLD signal (Lewis-Peacock and Postle, 2012). They demonstrated that that putative “category-selective” brain regions showing elevated delayed period activity during the STM retention of one category of information nonetheless carried patterns of activity associated with *another* category of information (specifically, the category currently relevant for behavior).

The possibility that signal intensity-based and MVPA methods might provide qualitatively different conclusions about neural function invites reconsideration of studies relying on the former strategy. In particular, there is a long history of studies identifying dorsolateral prefrontal cortex and inferior parietal cortex as the representational substrates in STM, based largely on the observation of sustained, elevated signal during the delay period of STM tasks (as reviewed, e.g., in Curtis and D’Esposito, 2003; Todd and Marois, 2004). Recently, several studies have explicitly tested the representational capacity of brain regions exhibiting sustained, elevated delay period activity, and have shown that stimulus information (the stimuli were arrays of coherently moving dots) could not be decoded from the delay-period BOLD signal in such regions (Riggall and Postle, 2012; Emrich et al., 2013). In contrast, stimulus identity could be decoded from visual cortex and area MT+, regions which did not exhibit a sustained, elevated response during the delay period. These failures to decode stimulus identity from parietal and prefrontal cortex (Riggall and Postle, 2012; Emrich et al., 2013) leave open the question of what might be the function of the sustained, elevated activity observed during STM delay periods; they also call into question the interpretation of previous studies that relied on elevated activity to make inferences about representational states in STM (Griffin and Nobre, 2003; Nobre et al., 2004; Lepsien and Nobre, 2007; Nee and Jonides, 2008, 2011; Oztekin et al., 2010). We will next discuss several studies that have addressed this issue within the frameworks of Cowan, Oberauer and McElree, testing their assertions that information in STM can be retained in various states that differ according to the level of activation of LTM representations.

The first study (Lewis-Peacock et al., 2012) employed a dual-response STM task with retrocuing adapted from Oberauer (2005). Each trial began with the presentation of two stimuli drawn from different categories (the categories were pairs of line segments, pronounceable non-words, and words). After an initial delay period, a retrocue appeared to signal which of the two memory items would be the target of the first memory probe. A second delay period followed, during which subjects had to retain both the cued item and the uncued item in memory. Subjects were instructed to respond to a memory probe according to instructions designed to encourage different formats of STM retention for the three categories of stimuli: (1) subjects had to make an orientation judgment for line segments (“visual” STM); (2) a synonym judgment for words (“semantic” STM), and a rhyme judgment for pseudowords (“phonological” STM); and (3) These different criteria were selected to enable maximally sensitive decoding of the stimulus categories, especially important because items from two categories would need to be simultaneously retained. Both items had to be retained even in the delay period after the first retrocue, because on each trial, after the first memory probe, a second retrocue appeared that would indicate, with equal probability, that either the initially cued (on “cue-repeat” trials) or the initially uncued (on “cue-switch” trials) memory item would be relevant for the second memory probe. This manipulation created a situation during the delay period following the first cue in which two items had to be remembered, but only one item was prioritized by the retrocue. Based on Oberauer (2005) and our own (LaRocque et al., 2013) behavioral work, we assumed that the cued item was in the FoA (we refer to such an item as an “attended memory item”, or AMI). The uncued, but still retained, item was assumed to be held outside the FoA (an “unattended memory item”, or UMI). In Oberauer’s studies, the fate of the uncued memory set was to transition from the SDA into aLTM (Oberauer, 2005; Oberauer and Hein, 2012). Therefore, we consider the UMI in our studies to also be in aLTM.

**Figure 2:**
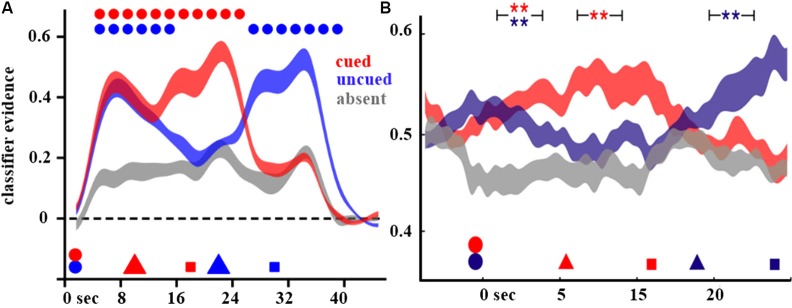
**Decoding of attended and UMIs**. Shown are the decoding results from Lewis-Peacock et al. (2012) **(A)** and LaRocque et al. (2013) **(B)** on cue-switch trials. In both figures, classifier evidence for the stimulus category is plotted as a function of time, with the timing of events in the trial indicated by geometric shapes on the *x*-axis (circles indicate stimulus presentation, triangles indicate the cues, and squares indicate the memory probes). The color scheme indicates the categories with respect to the cues: cued (red) is the category indicated by the first cue, uncued (blue) is the category of the other stimulus which is not selected by the first cue, and absent (gray) is the category not present on that trial.

In order to separately assay the neural representations of the two memory items, a pattern classification approach was used. The pattern classifiers were trained on a simple one-item STM task using stimuli drawn from the same three categories. After validating the classifiers’ performance at distinguishing the single-item trials with line segments vs. non-words vs. words, the classifiers were then applied to neural activity from the two-item retrocuing task. The dependent measure of interest was the classifier evidence for each of the three categories. Mathematically, it is the inner product of the feature weights (fixed by training on the one-item task data) by the feature values (the *z*-scored BOLD signal in corresponding voxels from the two-item task). Classifier evidence reflects the correspondence of the new brain data with the distinct patterns of brain data on which the classifier was trained; thus, it can be construed as an estimate of the presence of the pattern of neural activity specific to the category of interest. In the initial delay period, before any cues were presented, classifier evidence for the categories of the two items presented on each trial was significantly higher than the evidence for the non-present category (i.e., the classifier’s evidence for the stimulus category not presented on each trial served as a trial-by-trial baseline). After the appearance of the first retrocue, evidence for the AMI’s category remained high, but evidence for the UMI’s category dropped to baseline (Figure 2A). On cue-repeat trials, this pattern was reproduced following the second retrocue. However, on cue-switch trials, the strong evidence for the initially cued category dropped quickly to baseline and the classifier evidence for the newly cued category rose precipitously and remained elevated for the duration of the final delay period. These results suggest that an active neural representation is only present for items in STM when they are potentially relevant for a behavioral response, and thus putatively held in the FoA. Provocatively, the results also suggest that UMIs, although presumed to be in aLTM, may not be maintained via active neural representations, though evidence for their active neural representation can be restored when UMIs are cued to become AMIs.

One important caveat about the results of Lewis-Peacock et al. (2012) is that they are derived from the BOLD signal. It is possible, however, that UMIs may be retained in an active state to which the BOLD signal is insensitive. This objection is not merely theoretical; recent studies have shown, for example, that in the same individuals performing the same task, the BOLD signal can be shown to be insensitive to perceptually suppressed stimuli to which high-frequency neural oscillations are sensitive (Maier et al., 2008). Indeed, many studies have shown that neural oscillations across populations of neurons are sensitive to the short-term retention of information (Jensen and Tesche, 2002; Fuentemilla et al., 2010; Simanova et al., 2010). Therefore, we sought to replicate the important features of the Lewis-Peacock et al. (2012) study while assaying neural activity with EEG, a technique sensitive to neural oscillations. This study was also notable for its attempt to decode stimulus information from delay-period oscillatory neural activity, a challenging design which is only slowly being adopted (for a recent review, see Jafarpour et al., 2013). The classifiers used in this study were trained and tested on frequency-transformed EEG data, allowing us to assay the information present in patterns of oscillatory signals. In this conceptual replication, evidence for active neural representations was found for AMIs but not for UMIs; classifier evidence for the latter was indistinguishable from baseline (Figure 2B). However, when a UMI was cued as relevant for the next memory probe, its active neural activity pattern was reinstated and, behaviorally, subjects were able to respond accurately to probes of these items.

With the higher temporal resolution afforded by EEG, we were also able to estimate a time-course of the loss of classifier evidence (i.e., evidence for an active neural representation) for the UMI following the retrocue. By approximately 1.25 s after the onset of the retrocue, evidence for the UMI was indistinguishable from baseline. This provides a neural counterpart to behavioral evidence suggesting that approximately 1 s is needed between the retrocue and the memory probe in order to observe the RT benefit of a retrocue (LaRocque et al., 2013). These metrics can be construed as characterizing the time needed for an item to transition from the FoA into a less prioritized STM state, such as aLTM (Oberauer, 2005).

Another important analysis was performed in the EEG dataset to address the possibility that UMIs may be accompanied by a neural representation different from AMIs, but equally active in a neurophysiological sense (LaRocque et al., 2013). After all, a potential objection to our strategy of training classifiers on a one-item memory task before using them to assay AMIs and UMIs is that, in the one-item task, attention was not explicitly controlled. Subjects were therefore free to (and probably did) allocate attention to the one item being retained, in which case the item would be considered an AMI. If AMIs and UMIs are indeed maintained in a qualitatively different manner, then it follows that a classifier trained on AMIs might fail to detect UMIs. Therefore, we performed a separate classification analysis using only data from the two-item task to test this idea. We trained a classifier to predict, on each trial, whether a line segment stimulus was present (the line segment category was used because it was the one for which classification was most accurate, and therefore afforded the greatest sensitivity), separately for trials when a line segments were AMIs and when they were UMIs. We could only correctly classify the presence of line segment stimuli when they were AMIs. This indicated that there was no reliable difference between delay-period activity patterns from trials with line segments as a UMI and delay period activity patterns from trials in which no line segments had been presented; no evidence was found for an “alternative”, but active, form of neural representation for UMIs.

In considering the results of the Emrich et al. (2013) and Lewis-Peacock et al. (2012) studies, one might wonder if there is a contradiction in the MVPA results. In the former study, we found changes in classifier evidence that correlated with the precision of subjects’ memory, whereas in the latter we found a binary distinction, in that category-level classifier evidence was present only for AMIs, and not for UMIs. We believe that these results do not conflict because of the difference between category- and item-level decoding. On the one hand, classifier evidence obtained from decoding at the item level reflects quality of a stimulus representation. However, it is unclear if such a result should be expected to hold for category-level decoding. Consider, for example, the case for a trial in which a subject has a slightly inaccurate memory, and therefore an imprecise representation in STM, of the meaning of a word; would that affect the extent to which a word’s meaning-specific pattern of neural activity would be present, as opposed to line segment- and pseudoword-specific patterns? It seems possible that having a semantic representation in STM, whether it is precise or imprecise, could produce strong category-level patterns of bran activity. The broader concern still exists, however: what is the difference between decoding at the item level and at the category level? On the one hand, both approaches should assay active neural representations, because the neural representation of any given memory item should differ both from the representations of other within-category items and from items in different categories. If either of these distinctions were absent, then it would be difficult to argue that stimulus information was being actively represented. However, there are likely many more factors differentiating the cognitive states associated with remembering items from two different categories than remembering items from within the same category. Taking houses and faces as an example, it is easy to convince oneself that there may be differences in the cognitive processing accompanying memory for faces as compared to houses; e.g., if a house makes one think of real estate prices because of an impending home purchase, whereas an image of a staring face reminds one of celebrity mug shots seen on the cover of a trashy periodical, then neural activity predicting the retention of a face vs. a house in memory may be due to these accessory details rather than the stimulus properties. This is, of course, a general problem of psychological experiments. Completely controlling the cognitive activity of a subject is impossible. This does suggest, however, that item-level decoding of BOLD signal is likely to be successful in a more specific set of brain areas than category-level decoding. It is difficult to imagine that there are different emotional saliencies associated with, e.g., dots moving 37° vs. 157° clockwise of vertical.

In the final study we will consider that explicitly sought neural correlates of representational states in STM (Nelissen et al., 2013), subjects were presented with two items to remember on each trial. These items were images drawn from different categories, which included faces, bodies, and houses. After a brief initial delay, subjects were provided a cue indicating which of the two items would be relevant for an upcoming recognition probe array. The BOLD signal following this cue was decoded using classifiers trained on a visual perception condition in which subjects viewed images drawn from the same three categories. The authors found that in occipitotemporal cortex, the evidence for the cued category remained above baseline following the cue, while evidence for the uncued category (the UMI) dropped, in agreement with our results (Lewis-Peacock et al., 2012; LaRocque et al., 2013). Intriguingly, the UMI evidence in Nelissen et al. (2013) actually dropped *below* the baseline represented by the classifier’s evidence for the non-present category. However, a crucial detail is that subjects were able to completely forget the uncued memory item, because only a single probe display, targeting the cued item, appeared on each trial. Additionally, the uncued item’s category was present in the probe array, perhaps encouraging subjects to adopt a strategy of active suppression. Nonetheless, the overall pattern of results, obtained from a classifier trained on stimulus perception trials, is supportive of the primary role of attention in determining whether the contents of STM can be decoded from delay-period neural activity, and therefore whether there is evidence for the active neural representation of items in STM.

### Short-term memory (STM) and visual search

The cognitive construct of STM overlaps extensively with other cognitive constructs; indeed, it has been argued that some very transient form of STM is necessary even for the continuity of conscious experience (Edelman, 1989). One important area of overlap is with the construct of visual search. Both STM and visual search studies typically use behavioral paradigms that require subjects to transiently retain information about a stimulus in order to recognize and/or respond to that stimulus when it is re-presented at some later time. Visual search tasks often entail the detection of a memory item in an array of many (>4) new probe items. Although different terminology may be used (STM items vs. search templates), the behavioral phenomena being studied in the STM tasks and the visual search tasks are arguably very similar. This similarity was recently highlighted by the discovery of contralateral delay activity (CDA)—an electrophysiological marker that has been observed with the short-term retention of lateralized visual information—during the performance of visual search tasks (Emrich et al., 2009; Carlisle et al., 2011). The CDA is the difference in voltage between posterior electrodes contra- and ipsilateral to the visual field in which the memory items were presented. The magnitude of the CDA reflects the number of items being held in STM (up to an individual’s memory capacity), and it seems to be sustained throughout the delay period of STM tasks (Vogel et al., 2005). In the Carlisle et al. (2011) study, a CDA was observed when a search target was presented to one side of central fixation. With each successive trial searching for the same target stimulus, the magnitude of the CDA *decreased*. The authors interpreted this as the offloading of an “attentional template” from WM to LTM. This result is consistent with our own finding that active neural representations of UMIs are not evident in either fMRI or EEG recordings, and it raises the question of whether an offloaded “attentional template” differs in any meaningful way from our construct of an “UMI”. The suggestion that LTM resources can be used to support retention of an item outside WM is similar to the notion that items outside the SDA rely on activated LTM. However, we suggest that the presence or absence of above-threshold neural signals should not be used as the criterion for considering a process to recruit “STM” or “LTM” resources. We will return to this point later when we discuss alternate mechanisms of short-term retention besides one relying on elevated neural activity.

The Carlisle et al. (2011) result provides an opportunity to more deeply consider several issues. We have trumpeted the advantages of using a multivariate approach to identify stimulus-specific signals when attempting to measure active neural representations. In considering the Carlisle et al. (2011) results, the non-specificity of the CDA makes it difficult to interpret a reduction in its magnitude. Is the CDA a marker of STM retention? Or does it track active neural representations, which we posit are only present for STM items currently in the FoA? Though there are data inconsistent with some non-mnemonic interpretations of the CDA (Ikkai et al., 2010), the CDA has been shown to reflect object-tracking (Drew and Vogel, 2008) and to have a higher magnitude in an object-tracking condition vs. a memory condition for those same objects’ starting locations (Drew et al., 2011). These latter two results suggest that the CDA may index demands placed on attention. Yet another interpretation is suggested by results from a recent WM training study. In this study, participants completed daily cognitive training on an N-back WM task. After 5 weeks of training, subjects showed improved performance on the task, and this improvement was accompanied by a *reduced* CDA magnitude on both visual STM and visual search tasks compared to a pre-training experiment (Kundu et al., 2013). This result suggests that a reduction in the amplitude of the CDA may reflect increased efficiency of stimulus coding. The data are less consistent with an interpretation in which the reduction in CDA is accounted for by the increased utilization of LTM, because the memory items were not repeated across consecutive trials as they were in Carlisle et al. (2011). Therefore, it may be premature to suggest that a reduction of the CDA signifies the removal of an attentional template from WM. One way of adjudicating among the various interpretations of the reduction in CDA seen in Carlisle et al. (2011) would be to apply pattern classifiers to those data to test whether the classifier’s assessment of the strength of the attentional template would decrease in lockstep with the shrinking CDA. If so, this would provide confirmatory evidence that the active neural representation of the search template is being shifted to a less active state in memory, perhaps one relying more on LTM resources and less on attentional or STM resources.

Another visual search study has addressed the question of whether the neural representations of memory items remained active when those items were no longer in the FoA (Peters et al., 2012). In this study, subjects were presented with two stimuli, which were always pictures of faces or houses. After a brief delay, a retrocue indicated which of the two stimuli would be relevant for an upcoming serial search task. In this task, merged transparent images, each containing overlaid face and house images, were presented rapidly; the task was to indicate with a button press if one of the images exactly matched the stimulus that had been indicated by the retrocue. Note that, for successful task performance, the uncued memory item still had to be remembered even though it was irrelevant for the first visual search, because it would become the search template for a subsequent stream of images. In contrast to the absence of evidence for active representation of a UMI as reported in the Lewis-Peacock et al. (2012) study, decoding of the category of the irrelevant memory item was successful in no less than 17 distinct clusters of voxels. Upon first consideration, this result might seem to be problematic for our account that UMIs are not accompanied by an active trace. There are, however, a few methodological differences that may account for the disparate results. The first is that in the Peters et al. (2012) study, the UMI *always* became relevant for the second half of each trial; in our own experiments, the UMI subsequently became relevant on only half of the trials. The second methodological difference is that, in the Peters et al. (2012) study, the uncued memory item actually appeared in the search stream on half of the trials, raising the possibility that subjects may have adopted a strategy of actively suppressing the representation of the uncued memory item in order to prevent false alarm responses to this item. Active suppression of information is arguably different from passive retention of information (Luck and Sawaki, 2011), making it difficult to interpret their decoding results as reflective of the retention state of UMIs. More work is needed to understand how active suppression and the certainty of a UMI’s relevance might affect active neural representations for a transiently unattended memory item.

## Discussion

### Representational states in short-term memory (STM), reprised

We have considered several prominent theoretical models of STM, several behavioral and neurophysiological studies motivated by those models, and a series of studies employing MVPA to more directly assay neural representations in STM. The primary finding from MVPA studies from our group (Lewis-Peacock et al., 2012) and LaRocque et al. (2013) is that active neural representations are present for items in the FoA, but not for items in aLTM. This result confirms the strong distinction drawn by all three STM models between information selected by the FoA and information being retained outside the FoA (Cowan, 1988; McElree, 1998; Oberauer, 2002).

We now turn our attention to points on which the three theoretical models diverge. Highly sensitive multivariate analyses of EEG and fMRI measures of neural activity found no evidence for active neural representations of UMIs (Lewis-Peacock et al., 2012; LaRocque et al., 2013). This result seems to be at odds with the proposal that there is a state in STM with intermediate levels of activation (i.e., aLTM) between the FoA and the massive network of latent LTM (Cowan, 1988; Oberauer, 2002). However, we do not suggest that this result falsifies those models; rather, we propose that this result should be taken as a call for clarification of what is meant by “activated” as used by these theoretical accounts of STM. The original formulation was intended to demarcate STM from the immense network of latent LTM representations, but based on the results reviewed here, cognitive activation described in these models does not map directly onto neural activation. Rather than referring to such items as being retained in the “activated” portion of LTM, a word more in keeping with the function of STM—such as “accessible”—might be more appropriate to describe STM items outside the FoA. Crucially, we do not take these results as evidence for an isomorphism between aLTM and latent LTM, as suggested by the McElree (2006) model. We will return to this point momentarily, when we discuss possible neurophysiological mechanisms for the retention of UMIs.

An important question to address is whether the results reviewed here support the distinction drawn by Oberauer between the FoA and an intermediate SDA. The probe-based neuroimaging studies of Nee and Jonides (2008, 2011, 2013) do support a distinction in the retrieval-associated neural activity associated with memory probes to items in those two states (but see Oztekin et al., 2010). However, a description of the activation level of these neural representations during the retention interval is still wanting. In our retrocuing paradigm, subjects only retained one item outside the FoA, the UMI, which was presumably in aLTM (Lewis-Peacock et al., 2012; LaRocque et al., 2013). Therefore, we cannot conclusively address the activation level of neural representations in the SDA.

While we cannot conclusively speak to the activation level of the SDA, the study of Emrich et al. (2013) does provide a hint, in that evidence for active neural representations was lower (but still above baseline) as the STM load increased from one to three. Though this study did not explicitly control for the allocation of attention, the presence of an intermediate level of evidence for high-load memory items can be taken as consistent with the existence of an intermediate-activation-level SDA in which items in the high-load condition were retained. However, because the allocation of attention was not precisely controlled, another possibility is that all three items were in the FoA simultaneously. While we cannot conclusively adjudicate between these two interpretations, either one would contradict the one-item FoA posited by McElree’s model. (Note, however, that recently McElree’s studies have allowed that the FoA may not be limited to one item on tasks in which the items are presented simultaneously (Oztekin et al., 2010)). Indeed, the decoding results from Lewis-Peacock et al. (2012) and LaRocque et al. (2013) also contradict a one-item FoA, in that before the retrocue, there is evidence in the trial-averaged results for simultaneous active neural representations of both items in memory.

The absence of evidence for active neural representations of UMIs is consistent with one aspect of McElree’s model, in that he characterizes all memory representations outside the FoA as existing in a passive state of retention (McElree, 2006). While we appreciate his suggestion of a common storage substrate for aLTM and latent LTM, we still question McElree’s suggestion that the state of the information in aLTM is qualitatively identical (aside from varying memory strength) to all other LTM items. To encapsulate our objection with a concrete example, it seems unlikely that the seconds-old neural representation of the first word presented in a list of words is in the same state as the LTM representation the subject may have of the salutation the experimenter used in greeting her 30 min earlier. In particular, we note that decades of neuropsychological studies of patients with anterograde amnesia (which can often be secondary to MTL damage) dissociate those two states of memory. Patients with anterograde amnesia are able to remember recently presented items from a list, so long as that list does not exceed their STM span. They would not, however, be able to remember a greeting they had received 30 min previously, which a healthy control subject would have no trouble recalling. This is because the latter example requires encoding the greeting as an episodic memory in LTM, whereas briefly remembering a list of words does not. Typically, the intact STM in anterograde amnesics has been explained by an active rehearsal or refreshing mechanism; but this account suggests a state for the information that is readily refreshable, as conceptualized in Oberauer’s SDA (Oberauer, 2005). [We note that much recent research has shown that STM (and, indeed, perception) can be shown to be compromised in patients with MTL lesions in certain behavioral paradigms, especially those with novel or complex stimuli (Olsen et al., 2012; Rose et al., 2012; Yonelinas, 2013). However, the basic finding that patients with MTL lesions have relatively unimpaired STM coexisting with near-complete anterograde amnesia is still intact].

The previous example of a patient with anterograde amnesia brings up an issue very germane for discussions of STM and LTM: what is the role of MTL in STM? The literature addressing this topic is large and evolving, but it seems clear that the long-standing notion that the MTL is only involved in LTM is an over-simplification. Especially notable is that distraction during a short retention interval can interfere with the performance of patients with MTL lesions on STM tasks (Vargha-Khadem et al., 1997; Rose et al., 2012). Integrating these results into the framework of the STM models we have discussed hinges on the theoretical interpretation of the distractor’s effect in those paradigms. One interpretation is that the FoA automatically selects the distracting stimuli, thereby deselecting the memoranda. This interpretation would suggest that impairment on this task in patients with MTL damage is evidence for MTL involvement in the retention of information outside the FoA. Another interpretation emerges from recent work highlighting a critical role of MTL in the representation (both mnemonic and perceptual) of complex and/or novel stimuli, specifically in the binding of features within an item and the binding of items to their task contexts (for reviews, see Olsen et al., 2012; Yonelinas, 2013). Distraction may unmask this deficit by preventing rote rehearsal of the memoranda, regardless of whether the memoranda are in the FoA (e.g., if a research participant can use subarticulatory rehearsal to maintain verbal memoranda, there is no need to bind the memoranda to their context because they are the only items being actively retained *or* processed, and so there is little chance of a misbinding error). At this time, it is unclear if the sensitivity to distraction in patients with MTL damage should be construed as relating to a deficit in attention, binding, or both. One way to resolve this controversy would be to use a retrocuing task in a study of MTL lesion patients; a selective impairment in memory for UMIs relative to healthy controls would suggest a role for MTL in STM for items outside the FoA.

We have suggested that the gradually decreasing accessibility of less recently seen memory items need not correspond to an episodic, hippocampus-dependent memory state as suggested by McElree (McElree, 2006; Oztekin et al., 2010). Indeed, it seems more parsimonious to us to envision that there is a preexisting set of LTM representations which, after being selected and then deselected by the FoA, are transiently more accessible due to some property of the representation, without ever invoking the hippocampus or episodic retrieval. This mechanism could be “passive”, in the sense that it may not rely on heightened neural activity, yet be distinct from the mechanism of long-term potentiation thought to support LTM. This naturally raises the question: what sort of neurophysiological mechanism could support such a property?

### Neurophysiological correlates of short-term retention outside the focus of attention

The results of Lewis-Peacock et al. (2012) and LaRocque et al. (2013) cannot address the neurophysiological mechanism by which UMIs might be retained, if not by the canonical mechanism of reverberatory activity in a network of stimulus-sensitive neurons (Hebb, 1949). However, there are plausible alternative models of short-term retention that do not rely on elevated neural activity as the sole mechanism for information retention. First, recent work in mice has shown that knocking out the GluR1 subunit of the AMPA receptor results in a selective defect in STM (Sanderson et al., 2009). Slice electrophysiology experiments subsequently showed that this mutant also had a deficit in short-term potentiation (STP; Erickson et al., 2009). STP induction protocols are very brief (~60 ms), with potentiation onset within 2–3 s of induction, and produce synaptic potentiation that decays very quickly (on the order of a minute). All these properties make STP a plausible mechanism for the retention of UMIs. A similar proposal has been made regarding a transient elevation of calcium concentration in the presynaptic neuron, which also possesses the traits of rapid instantiation and a moderately slow decay that would be required for any mechanistic account of STM (Mongillo et al., 2008).

At the systems level, one model of STM-by-transient-synaptic-modification employs a “matched filter” mechanism whereby the information stored in a network of transiently potentiated synapses could be reinstated as a pattern of neural activity. In this scheme, a network of transiently potentiated synapses in inferior temporal cortex can act as a matched filter, thereby providing an enhanced neural response to inputs matching the input that first instantiated the synaptic weights (Sugase-Miyamoto et al., 2008). Evidence for a similar scheme also comes from a multivariate analysis of neuronal activity in prefrontal cortex (PFC), in which “implicit” changes in synaptic weights adaptively code moment-to-moment changes in behavioral context (Stokes et al., 2013). Notably, a mechanism such as this implies information storage within a network defined by a distributed pattern of synaptic weights, which is how LTM storage is typically understood. Although the neuroimaging data that we have reviewed in this report do not speak directly to the cellular- and systems-level models summarized in this and the preceding paragraph, we can nonetheless observe that our data are consistent with these types of mechanisms supporting the short-term retention of UMIs.

One question that has been posed to us repeatedly in discussing the results of LaRocque et al. (2013) and Lewis-Peacock et al. (2012) is some variant of the following: “How can you be sure that STM without an active neural representation isn’t really just LTM?”. We confess that this question is vexing, because it seems to emerge from circular definitions. STM has a distinct behavioral definition—it refers to the retention of information over a short delay in order to guide behavior. LTM also has a distinct behavioral definition—the long-term retention of information that is *not* presently being used to guide behavior. In our opinion, the terminology used to describe neurophysiological mechanisms should be clearly demarcated from the description of behavior, because there is no *prima facie* reason to mandate that separate mechanisms must be used to support retention across different intervals of time. Indeed, recent work has challenged the notion that purported “STM” and “LTM” mechanisms can be cleanly separated (Ranganath and Blumenfeld, 2005; Rose et al., 2012). Even if separate neural mechanisms are often observed supporting the retention of information across different intervals, the interpretation of such distinctions suffers by conflating the terminologies for behavior and neurophysiology. In discussing neurophysiological mechanisms of memory, we prefer to use language suggested by O’Reilly et al. (2012), and refer to “weight-based” and “activity-based” mechanisms. Storage based on synaptic modifications, like STP, is weight-based, whereas storage based on sustained neural firing, such as a Hebbian “reverberatory trace” (Hebb, 1949), is inherently activity-based. Therefore, to return to the question of how to categorize UMIs, we suggest that UMIs and AMIs are both “in” STM, where the retention of information may be accomplished via weight-based mechanisms for the former and via activity-based mechanisms for the latter.

Having considered neuroimaging studies of memory probe-evoked activity (Nee and Jonides, 2008, 2011, 2013; Oztekin et al., 2010), retrocue-evoked activity (Griffin and Nobre, 2003; Nobre et al., 2004; Lepsien and Nobre, 2007), and delay-period activity (Lepsien and Nobre, 2007; Lewis-Peacock et al., 2012; Trapp and Lepsien, 2012; Nelissen et al., 2013; Emrich et al., 2013; LaRocque et al., 2013), the weight of the evidence suggests that there are multiple representational states in STM. Specifically, we argue that the neural evidence is supportive of those models that posit the simultaneous, (neurophysiologically) active short-term retention of multiple items, either in a multi-item FoA (Cowan, 1995) or an SDA (Oberauer, 2002). The studies we have reviewed also suggest a constraint for theoretical models of STM; namely, that STM, *per se*, may not require elevated neural activity, and that attention is the primary determinant of the presence of neurophysiologically active representations in STM.

### Attention and retention: limitations of the present work and directions for the future

In the course of our discussion of attention and states in STM, we have highlighted results obtained from MVPA of neural data. Though we have argued for the increased sensitivity and inferential power of MVPA compared to traditional signal-intensity-based analyses, we note that MVPA has several important limitations. First, it is limited by the type of neural data used to train the classifier. MVPA applied to fMRI or EEG measures of neural activity can glean information from subtle patterns of neural activity, but it is insensitive to processes that are not manifested in EEG or fMRI measures. For example, a classifier trained on BOLD signal would be insensitive to information contained in inter-region patterns of connectivity or in a spike-timing-dependent neural code (though classifiers could certainly be trained on those data). A second potential limitation of MVPA is the possibility that the patterns of neural activity measured by fMRI and EEG are epiphenomenal, and do not directly assay the neural representations that are important in the guidance of behavior. Though this is an important scenario to keep in mind, we consider it to be unlikely, especially because of the results we have discussed that show correlations between patterns of neural activity in sensory cortex and task performance (Emrich et al., 2013; Ester et al., 2013). A potential topic for future study would be to test the relationship of MVPA evidence to behavior by perturbing patterns of neural activity with a perturbational method, such as transcranial magnetic stimulation, and observing the resultant behavioral effects. For further reviews of the limitations of MVPA as applied to neural data, see Tong and Pratte (2012) and Davis and Poldrack (2013).

One of the intriguing suggestions of the neuroimaging studies of STM with retrocues reviewed above is that the neural activity observed during the delay period of a STM task may be more related to the allocation of attention to information, rather than to the retention of information, *per se*. In order to reach this conclusion, it was important to explicitly control the allocation of attention. It follows from this that many studies of STM that lack such explicit control of attention may have inadvertently conflated short-term retention with attention. Many studies have recently ascribed properties to STM that might in fact relate more closely to the FoA. Examples of such conclusions from the recent literature include: the contents of STM alter the detection thresholds of memory-matching stimuli and the size of a neurophysiological marker of visual awareness, the P300 (Melloni et al., 2011); STM can influence perception by shifting the perceived direction of motion of a moving stimulus away from a remembered direction (motion repulsion) (Kang et al., 2011); and information retained in STM can influence the perception of an ambiguous stimulus (Scocchia et al., 2013). In each of these cases, the items retained in STM were also presumably in the FoA, raising the possibility that the properties ascribed to STM may more directly relate to attention. A potentially fruitful topic for further study will be the disambiguation of properties of STM and attention applied to items in STM.

We have defined AMIs and UMIs by the differential allocation of attention to information in STM. In the study of consciousness, a topic of ongoing debate is how the allocation of attention relates to conscious awareness. This debate led us to wonder about the status of AMIs and UMIs with respect to conscious awareness. Except for certain conditions of perceptual suppression (e.g., with binocular rivalry), information selected by the FoA typically becomes the content of consciousness (see, e.g., Table 1 in Koch and Tsuchiya, 2006). A more tangled question is how information in STM, but not currently selected by the FoA, might relate to conscious awareness. To begin untangling this question, we should note that, though we have called the memory items “unattended”, the use of this term is intended to draw contrast with the unequivocally prioritized and attended item that was cued in the retrocuing tasks (Lewis-Peacock et al., 2012; LaRocque et al., 2013). It could indeed be the case that no attention is allocated to UMIs. Alternatively, it could be that attention is periodically, briefly allocated, or even that a very small amount of attention is continuously allocated to “UMIs”. Finding evidence to distinguish these possibilities is empirically possible, albeit challenging. Each of the three scenarios could be true for different subjects, or across different trials for a single subject. This difficulty in conclusively describing items purportedly outside the FoA mirrors an ongoing debate in the study of visual awareness as to whether it is possible to be conscious of a stimulus with no attention allocated to that stimulus; in that literature, it has remained hotly debated because of the difficulty inherent in creating a “no-attention” condition (Koch and Tsuchiya, 2006; Van Boxtel et al., 2010; Cohen et al., 2011). STM items in the FoA can be considered to be isomorphic with the contents of consciousness (Baars and Franklin, 2003; Buchsbaum, 2013). If UMIs are indeed outside the FoA, perhaps they can also be considered to be transiently outside conscious awareness, though still consciously accessible. After all, the behavioral criterion for STM is that it should be consciously accessible after a delay period (but see Soto et al., 2011 for a possible example of STM without conscious awareness). Another possibility is that UMIs may be in conscious awareness, and that they therefore represent another dissociation of attention and awareness. These distinctions between attention and awareness are important, and they could provide a fruitful way to understand the various states of transiently retained information. Our findings point to the importance of controlling attention in STM tasks. Closer control of attention and awareness during tests of STM could provide a means to productively address the problem of states of representation in STM, and to build models that more comprehensively encompass the theoretical and the neural mechanisms underlying and supporting STM.

## Conflict of interest statement

The authors declare that the research was conducted in the absence of any commercial or financial relationships that could be construed as a potential conflict of interest.
